# The Use of Compression Stockings to Reduce Water Retention in the Legs During Gaming and Esports: Randomized Controlled Field Study

**DOI:** 10.2196/25886

**Published:** 2022-09-29

**Authors:** Steffen Christian Ekkehard Schmidt, Stefan Sell, Alexander Woll

**Affiliations:** 1 Institute of Sports and Sports Science Karlsruhe Institute of Technology Karlsruhe Germany; 2 Joint Center Black Forest Hospital Neuenbuerg Neuenbuerg Germany

**Keywords:** esport, streaming, gaming, water retention, fluid balance, compression stockings, bioelectrical impedance, mobile phone

## Abstract

**Background:**

With the increasing digitalization of daily life, internet-based entertainment such as gaming and streaming has advanced to one of the megatrends of the 21st century. Besides offering a multitude of controversially discussed opportunities for entertainment and social interaction, there is reasonable concern about health issues caused by the absence of physical activity among activities linked to gaming and streaming.

**Objective:**

The aim of this study is to compare the water balance of recreational gamers with and those without compression stockings during a gaming event.

**Methods:**

We measured body composition and water balance with 8-electrode bioelectrical impedance analysis among 46 recreational gamers with an average age of 27.1 (SD 6.5) years (5/46, 11% women and 41/46, 89% men) before and after 24 hours at a gaming event. Of the 46 gamers, 23 (50%) gamers wore compression stockings for the duration of the study.

**Results:**

Our study shows that prolonged gaming and associated behaviors during a 24-hour time frame lead to an increase in total body water (+0.76 L; *P*<.001) and a decrease of phase angle in the lower extremities (−0.47°; *P*<.001) but not in the upper extremities (+0.09°; *P*=.80), when no compression is used. Gamers using compression socks did not show any significant negative effects on their body composition.

**Conclusions:**

Prolonged gaming and streaming are serious risk factors for diseases associated with water retention in the legs, and these risks can be measured by bioelectrical impedance and reduced by wearing compression stockings. We conclude that these findings should be discussed and replicated in larger studies and that there is a considerably large market for compression stockings among gamers and live streamers.

## Introduction

### Background

A multitude of diseases caused by physical inactivity is well-documented [1-3]. It is also known that physical activity (PA) reduces the association of sitting time with mortality [4], and an active lifestyle during childhood reduces the risk for several diseases in adulthood [5]. Therefore, the World Health Organization promotes PA as part of a healthy lifestyle [6]. Among youth, recent epidemiological studies point to stagnation at insufficient amounts of PA [7-9], paralleled by a global increase in screen time [10], and reviews and meta-analyses proclaim a global *PA crisis* with only approximately 25% of youth reaching the PA guidelines by the World Health Organization [7,8]. Recent studies show that the absence of PA and the factors associated with intensive media use are independent risk factors for a variety of different nonspecific (eg, overweight and metabolic syndrome) and specific [11-15] diseases.

Besides the aforementioned long-term consequences and risks of physical inactivity, negative short-term consequences from even a single event of prolonged physical inactivity have been confirmed by experimental studies [16]. One disease that is discussed to be aligned with single periods of continuous physical inactivity is thrombosis of the deep leg veins or venous thromboembolism (VTE) as initially described by Homans in 1954 [17]. This disease was specifically found after long air travels in the 1980s and therefore initially called the *economy class syndrome* [18,19]. In the 2000s, similar clinical scenarios were found among professions with prolonged sitting or standing time, which were described as the *seated immobility thromboembolism* (SIT) in various studies [20-24]. SIT is directly correlated to physical inactivity and is even worsened by other recently common risk factors such as obesity and low levels of general PA [25]. With the increasing relevance of screen media in occupational and recreational lifestyle, thrombosis caused by intensive use of computers and the internet was given the new name *e-thrombosis* in 2003 by Beasley et al [26] as they described a case report about a 32-year-old man, in whom immobility associated with sitting for long periods while using a computer caused life-threatening VTE [26]. As computers became increasingly popular in the recreational setting and as new professions involving prolonged gaming arose, the topic gained interest, and the authors stated that gaming may be the 21st century variant of seated immobility [27,28].

Although heavily described as a risk factor [25-36], only a few studies tried to identify the driving or causal factors that lead to VTE during recreational use of screen time, and to the best of our knowledge, studies describing SIT or e-thrombosis in the context of gaming-related live streaming do not exist.

In a pilot study in 2017, we could show that 1 day of intensive gaming results in a significant increase in the amount of water in the lower extremities, paralleled by a significant and meaningful decrease in local and total body phase angle (PhA) measured by bioelectrical impedance analysis (BIA) [37]. Therefore, we returned to the event for the following 2 years and conducted a randomized controlled field study to measure the bioelectrical impedance of a total of 50 (n=46 complete data sets) male and female gamers with and without compression stockings before and after 1 day at the event.

### Objective

In this paper, we present the results of 46 gamers over 3 study years and discuss the effect of compression stockings on different parameters of body composition measured by BIA. The study is defined as a field study, and we did not control for the behavior of the gamers (eg, net amount of gaming, eating, or sleeping patterns). This study aims to clarify whether this population is at risk during their normal behavior and whether this risk can be measured by BIA and reduced by compression.

Our hypotheses were the following:

1. Prolonged gaming and associated behaviors cause an increase in total body water (TBW), which can be measured by BIA.
2. Prolonged gaming and associated behaviors cause an increase in body water as measured by a decrease in local bioelectrical tissue resistance (R) in the lower extremities but not the upper extremities.
3. Prolonged gaming and associated behaviors cause a decrease in the bioelectrical PhA in the lower extremities, which indicates an overall stronger pronounced increase in extracellular fluids compared with intracellular fluids.
4. Prolonged gaming and associated behaviors lead to an increase in extracellular body mass (ECM) but not in intracellular body mass (ICM).
5. The use of compression stockings can reduce the decrease in PhA and the increase in TBW.

## Methods

### Sample

**Figure 1:**
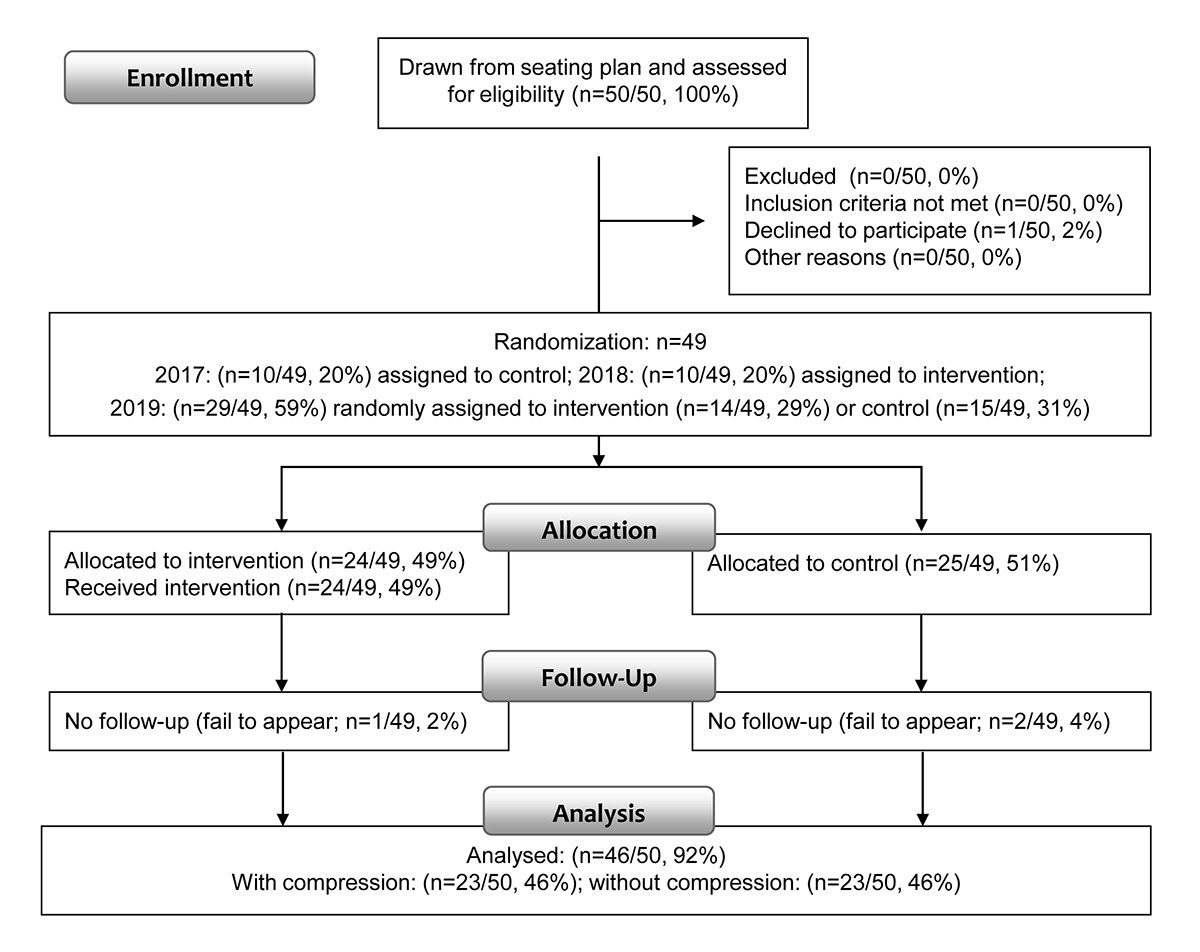
Recruitment process and design of the study.

The data were collected on gaming conventions in a rural area in Germany in May 2017, April 2018, and May 2019, hosted by a local computer club. The convention houses 400-430 participants annually and is one of the oldest gaming conventions in Germany, starting in the 1990s. Participants were recruited randomly using dice to select invitations from the 40×10 places large public seating plan. In 2017, a total of 20% (10/50) of the final participants were selected and received no compression. In 2018, a total of 20% (10/50) of participants were selected and all of them received compression. In 2019, a total of 60% (30/50) of participants were selected and alternately allocated to either the group with compression (15/30, 50%) or the group without compression (15/30, 50%). A total of 46 participants with a mean age of 27.2 (SD 6.9) years (5/46, 11% women and 41/46, 89% men) participated in both the pretest and posttest assessment and were included in the final sample (Figure 1). Participants without compression stockings are referred to as the control group (CG), whereas participants with compression stockings are referred to as the intervention group (IG). Participants agreed to participate in the study and received a US $23 cash incentive on completing the tests. Eligibility criteria included being a registered participant of the convention; that is, having a number on the seating plan; no current medication; and not being under the influence of legal drugs. All participants answered the questionnaires in the presence of a qualified interviewer on site. Participants were not informed about the research question or possible results of the study.

### Procedure

#### Overview

We used the same devices, questionnaires, and electrodes every year, and similar weather conditions occurred. The convention always started on Friday and ended on Sunday. The pretest assessment was hosted on Friday from 6 PM until midnight, and participants joined the posttest assessment after 24 hours, with a maximal deviation of 90 minutes.

Participants in the IG received a pair of VenoTrain cocoon (Bauerfeind) after measuring body height and calf circumference to ensure the accuracy of fit. They also received a pair of special gloves to wear the stockings easily and were advised to wear the stockings until they go to bed and wear them again immediately after awakening until the second measurement in the evening. During their stay at the event, participants were free to engage in any kind of activity they wanted. All participants brought their computers and played casually or in tournaments during the event. A participant reported having live-streamed his gaming during the event.

#### Anthropometry, Complaints, PA, and Media Use

Body weight was measured to the nearest 0.1 kg with a calibrated scale (seca) and standing height was measured to the nearest 0.5 cm using a stadiometer (seca), with the participant wearing light clothes and no shoes. BMI was calculated using the following formula:

BMI=weight (kg)/height (m)^2^ **(1)**

Participants were asked about the average time (minutes per day) on a normal week they spend with gaming on a smartphone, tablet, computer, or console; their total average daily screen time; active sports club membership (yes or no); and the days per week with at least 60 minutes of moderate to vigorous PA [38]. Recreational smartphone use was excluded from total sedentary screen time because it is not explicitly accompanied by sitting or physical inactivity.

We also tracked complaints regarding the lower extremities using a standardized numeric pain rating scale with seven 10-point items before the first BIA and immediately after the last BIA: “How do you sense your legs regarding the following symptoms: heavy legs, swollen legs, tingling, tension, pain, itching, or muscle aching? 1=no complaints to 10=very strong complaints.” This scale was provided directly by the manufacturer Bauerfeind and is not published.

#### BIA Procedure

BIA was conducted by trained investigators according to the European Society for Clinical Nutrition and Metabolism guidelines for BIA in clinical practice [39]. All participants were nonpregnant and healthy, deﬁned as the absence of a clinical condition that could inﬂuence ﬂuid balance; for example, renal, endocrine, or myocardial disease, as ascertained by an interview. Fasting was not a precondition for study participation. An 8-electrode BIA measurement of R and bioelectric reactance (Xc) was taken at a ﬁxed frequency of 50 kHz between the right wrist and ankle, as well as the left wrist and ankle (standard placement of surface electrodes) with a body impedance analyzer (BIA 2000-S; Data Input) while the participants were in a supine position on a nonconductive surface, with no contact with external metal objects. Before the examination, participants laid quietly in a supine position for a minimum of 3 minutes. R and Xc were measured at a 10-second interval until no changes in R and Xc between 2 measurements were observed, and the last measurement was noted.

BIA uses R to estimate TBW and to derive fat-free mass and fat mass. Besides R, BIA also provides information about the Xc, which is only caused by living cells with intact membranes [40]. From Xc, information about the amount of intracellular ﬂuid can be derived, and this information was used to estimate the total body cell mass (BCM). In addition, the PhA as of 57.297×arctan (R/Xc) is reported. Fat-free mass, BCM, and fat mass were derived from formulas included in the NutriPlus software package by Data Input [41].

The supine, single frequency BIA using adhesive electrodes has a technical error of <0.5% [42] and a 24-hour retest reliability of *r*>0.82 with an intraclass correlation coefficient >0.96 [43]. With *r*=0.96, the validity of estimating BCM versus dual-energy x-ray absorptiometry is high [44].

### Statistics

All statistical tests were conducted using SPSS (version 25; IBM Corp). Statistical significance was set to *P*<.05 and repeated measurement analyses of variance (rmANOVAs) were used to detect significant differences between the changes among the IG and CG as of time×group interactions. Besides *F* and *P* values, effect sizes are reported through partial eta^2^ (p. *η*^2^).

### Ethical Considerations

The Karlsruhe Institute of Technology institutional review board (IRB) has declared that this study type does not warrant ethics application as per their guidelines [45]. All participants provided informed consent. According to the Declaration of Helsinki, no humans were harmed during this study [46].

## Results

### Sample Characteristics

The characteristics and anthropometrics of the participants in the IG and CG are shown in Table 1.

Both groups showed a comparable range of age except for 1 older participant in the CG. Mean weight gain during the event was +0.8 (SD 1.1) kg and translated into a 0.2-point increase in BMI. In addition to age and anthropometrics, we asked the participants about their engagement in sports and their average daily gaming and total sedentary screen time (Table 2).

**Table 1:** Sample characteristics (n=46).

Group	Values, n (%)	Age (years), mean (SD); range	Height (cm), mean (SD)	Weight (kg), mean (SD)		BMI, mean (SD)	
				0 hours	+24 hours	0 hours	+24 hours
IG^a^	23 (50)	25 (4.5); 18-34	178.6 (10.2)	82.8 (11.7)	83.4 (12)	26 (3.6)	26.2 (3.7)
CG^b^	23 (50)	29.1 (7.6); 20-57	178 (7.5)	82 (13.0)	83 (13.2)	25.9 (3.7)	26.2 (3.7)
All	46 (100)	27.1 (6.5); 18-57	178.3 (8.8)	82.4 (12.3)	83.2 (12.5)	26 (3.6)	26.2 (3.7)

^a^IG: intervention group (compression).

^b^CG: control group (no compression).

**Table 2:** Gaming, screen time, and physical activity (n=46).

Group	Values, n (%)	Gaming (hours/day), mean (SD)				Sedentary screen time^a^ (hours/day), mean (SD)		Active sports club membership, n (%)		Physical activity^b^ (days/week), mean (SD)
		Smartphone and tablet	Computer	Console						
IG^c^	23 (50)	0.5 (0.7)	2.5 (1.5)	0.07 (0.3)	5.9 (3.2)		7 (30)		2.6 (1.6)	
CG^d^	23 (50)	0.6 (0.8)	1.4 (0.7)	0.03 (0.1)	7.6 (3.9)		7 (30)		3.1 (31.6)	
All	46 (100)	0.5 (0.7)	1.9 (1.3)	0.05 (0.2)	6.7 (3.6)		14 (30)		2.9 (1.6)	

^a^Total business and recreational screen time (recreational smartphone use excluded).

^b^Days per week with at least 60 minutes of moderate to vigorous physical activity on a normal week.

^c^IG: intervention group (compression).

^d^CG: control group (no compression).

### Bioelectrical Impedance Analyses

Participants reported that they reach the PA guidelines for adults on 2.6 (IG) and 3.1 (CG) days of a normal week and 30% (7/23) of participants in both groups were active members of sports clubs. Total sedentary screen time was slightly higher in the CG, with an average of 6.7 (SD 3.6) hours per day for the total sample, including a total of approximately 2.5 hours of daily gaming. Table 3 shows the results of the pre-event and postevent whole-body BIA and the related time and time×group interactions.

R, Xc, and all the derived parameters remained stable in the IG (Table 3). Among the participants in the CG, R, Xc, and PhA significantly declined, whereas TBW significantly increased, and a 0.8-kg increase in ECM was close to statistical significance (*P*=.05). The rmANOVAs revealed significant time and group interactions among R, Xc, PhA, and TBW. Descriptive differences in the change of ECM between groups were on the edge of statistical significance (*F*_1,44_=3.56; *P*=.06; p. *η*^2^=0.075), with participants in the IG losing 0.2 (SD 1) kg and those in the CG gaining 0.8 (SD 2.1) kg during the event.

The segmental BIA analyses showed no significant effects for time or time×group interactions for the upper extremities (Table 4). For the lower extremities, significant effects of time and time×group interactions were found for all parameters. Participants without compression showed a mean decrease of −11.4 (SD 15.8) *Ω* for R and −3.1 (SD 2.8) *Ω* for Xc, resulting in a mean decrease of the PA by 0.47 (SD 0.28) units, whereas participants with compression showed a slight increase of R (mean +3.6, SD 10.7 *Ω*), Xc (mean +0.9, SD 2.1 *Ω*), and PA (mean +0.09, SD 0.37 units).

**Table 3:** Pregaming and postgaming assessment of the bioelectrical impedance analysis results (whole body).

Measure		IG^a^, mean (SD)	CG^b^, mean (SD)	ANOVA^c^ (time)							ANOVA (time×group)				
				*F* test (*df*)		*P* value		p. *η*^2^		*F* test (*df*)		*P* value		p. *η*^2^	
**R^d ^(*Ω*)**					0.86 (1,44)		.36		0.019		5.26 (1,44)		.02		0.107
	0 hours	515.6 (55.1)	491.2 (69.2)												
	+24 hours	520 (55.4)	481 (73.1)^e^												
**Xc^f^ (*Ω*)**					3.56 (1,44)		.06		0.075		13.63 (1,44)		<.001		0.237
	0 hours	61.3 (6.3)	58.4 (7.4)												
	+24 hours	62.3 (7)	55.3 (6.8)^e^												
**PhA^g^ (°)**					8.72 (1,44)		<.001		0.165		20.12 (1,44)		<.001		0.314
	0 hours	6.80 (0.43)	6.84 (0.84)												
	+24 hours	6.85 (0.45)	6.60 (0.81)^e^												
**TBW^h^ (L)**					4.2 (1,44)		.04		0.087		5.28 (1,44)		.02		0.107
	0 hours	44.8 (6.5)	46.5 (6.4)												
	+24 hours	44.8** **(6.6)	47.2 (6.7)^e^												
**ICM^i^ (kg)**					0.04 (1,44)		.94		0.001		0.23 (1,44)		.63		0.005
	0 hours	34 (5)	35.4 (6)												
	+24 hours	34.1 (4.9)	35.4 (6)												
**ECM^j^ (kg)**					1.79 (1,44)		.19		0.039		3.56 (1,44)		.06		0.075
	0 hours	27.3 (4.1)	28.3 (4.2)												
	+24 hours	27.1 (4.4)	29.1 (4.3)												

^a^IG: intervention group (compression).

^b^CG: control group (no compression).

^c^ANOVA: analysis of variance.

^d^R: resistance.

^e^Within-group post hoc *t* test (2-tailed) for the effect of time significant (*P*<.05).

^f^Xc: reactance.

^g^PhA: phase angle.

^h^TBW: total body water.

^i^ICM: intracellular mass.

^j^ECM: extracellular mass.

**Table 4:** Pregaming and postgaming assessment of bioelectrical impedance analysis results for extremities.

Extremity and measure				IC^a^, mean (SD)		CG^b^, mean (SD)		ANOVA^c^ (time)						ANOVA (time×group)				
								*F* test (*df*)		*P* value		p. *η*^2^		*F* test (*df*)		*P* value		p. *η*^2^
**Upper**																		
	**R^d^ (*Ω*)**							1.41 (1,44)		.24		0.031		0.05 (1,44)		.82		0.001
		0 hours	260.1 (27.6)		248.5 (45.4)													
		+24 hours	262 (29.6)		251.1 (49.2)													
	**Xc^e^ (*Ω*)**							0.27 (1,44)		.60		0.006		0.43 (1,44)		.51		0.010
		Pregaming	29.9 (3.2)		28.7 (2.9)													
		+24 hours	29.9 (3.6)		29 (3.2)													
	**PhA^f^ (°)**							0.35 (1,44)		.56		0.008		0.85 (1,44)		.36		0.019
		Pregaming	6.57 (0.48)		6.71 (0.89)													
		+24 hours	6.51 (0.51)		6.72 (0.88)													
**Lower**																		
	**R (*Ω*)**							3.85 (1,44)		.05		0.080		14.37 (1,44)		<.001		0.246
		Pregaming	234.6 (28.1)		224.5 (29.7)													
		+24 hours	238.2 (25)		213 (28.4)^g^													
	**Xc (*Ω*)**							9.35 (1,44)		.004		0.175		29.26 (1,44)		<.001		0.399
		Pregaming	29 (3.5)		27.4 (4.8)													
		+24 hours	29.9 (3.8)		24.2 (4.3)^g^													
	**PhA (°)**							15.81 (1,44)		<.001		0.264		33.17 (1,44)		<.001		0.430
		Pregaming	7.08 (0.55)		6.89 (0.85)													
		+24 hours	7.16 (0.54)		6.41 (0.81)^g^													

^a^IG: intervention group (compression).

^b^CG: control group (no compression).

^c^ANOVA: analysis of variance.

^d^R: resistance.

^e^Xc: reactance.

^f^PhA: phase angle.

^g^Within-group post hoc *t* test (2-tailed) for the effect of time significant (*P*<.05).

### Complaints

Participants’ complaints concerning their lower extremities directly before the first and after the last BIA measurements are shown in Table 5.

Participants without compression complained significantly more about heavy legs (*F*_1,34_=4.25; *P*=.04; p. *η*^2^=0.111) and swollen legs (*F*_1,34_=5.17; *P*<.001; p. *η*^2^=0.132) compared with participants with compression. Participants with compression reported significantly more itching (*F*_1,22_=4.33; *P*=.04; p. *η*^2^=0.113).

**Table 5:** Complaints regarding the legs directly before and after the bioelectrical impedance analysis measurements.

Complaint	IG^a^ (0 hours), mean (SD)	IG (+24 hours), mean difference	CG^b^ (0 hours), mean (SD)	CG (+24 hours), mean difference	rmANOVA^c^ (time×group)		
					*F* test (*df*)	*P* value^d^	p. *η*^2^
Heavy legs	1.1 (1.3)	+0.13	0.4 (0.9)	+1.46	4.25 (1,34)	.04	0.111
Swollen legs	0.9 (1.1)	+0.08	0.1 (0.3)	+1.53	5.17 (1,34)	.03	0.132
Tingling	1.2 (1.6)	+0.08	0.4 (0.8)	+0.15	0.02 (1,34)	.87	0.001
Tension	1.2 (1.1)	+0.13	0.8 (1.2)	+0.23	0.02 (1,34)	.87	0.001
Pain	1.4 (1.6)	−0.21	1.5 (2.4)	−0.53	0.24 (1,34)	.62	0.007
Itching	0.7 (0.8)	+0.56	0.8 (1.5)	−0.38	4.33 (1,34)	.04	0.113
Muscle aching	1.8 (2.2)	−0.87	0.8 (1.5)	−0.76	0.39 (1,34)	.84	0.001

^a^IG: intervention group (compression).

^b^CG: control group (no compression).

^c^rmANOVA: repeated measurement analysis of variance.

## Discussion

### Principal Findings

Our study shows that extensive screen time use by gamers can cause short-term effects on water balance and its allocation among body compartments, which can be measured by BIA and prevented by compression stockings. We have summarized findings with regard to our initial hypotheses in the subsequent sections.

#### Prolonged Gaming and Associated Behaviors Cause a Significant Increase in TBW, Which Can Be Measured by BIA

We found a significant increase of 0.76 (1.20) L in TBW among the participants without compression. Of the 23 participants, 14 (61%) participants without compression showed increased TBW during the course of the study (mean +1.4, SD 1.1 L), whereas 2 (9%) participants showed no changes and 7 (30%) participants showed decreased TBW (mean −0.3, SD 0.2 L).

#### Prolonged Gaming and Associated Behaviors Cause a Significant Increase in Body Water as Measured by a Decrease in Local R Among the Lower Extremities but Not the Upper Extremities

As there are no published methods for directly quantifying the water in body segments measured by the BIA that we used and as those that exist for other BIA methods depend heavily on a multitude of assumptions such as the resistivity of the blood [47], we relied on the raw data of R to compare the extremities. Particularly within a time frame of 24 hours, one can directly conclude the changes of the extracellular water (ECW) content of a tissue by its changes in bioelectrical R. In our setting, we found no significant increase in R in the upper extremities of participants in both groups. However, in the lower extremities, participants without compression showed a mean decrease in R of −11.4 (SD 15.8) *Ω*, whereas the participants in the IG showed a slight increase of +3.6 (SD 10.7) *Ω*. This is consistent with data from a pilot study on leg fluid accumulation during sitting that found an exponential-into-linear increase in fluid volume in the legs of 14 participants over 150 minutes [48].

#### Prolonged Gaming and Associated Behaviors Cause a Decrease in Bioelectrical PhA in the Lower Extremities, Which Indicates an Overall Stronger Pronounced Increase in Extracellular Fluids Compared With Intracellular Fluids

Our data confirm hypothesis 3, with a significant interaction between time and group for the PhA of the lower extremities (*F*_1,44_=33.17; *P*<.001; p. *η*^2^=0.430) and the whole body (*F*_1,44_=20.12; *P*<.001; p. *η*^2^=0.314). The participants in the CG showed a decrease in their mean PhA by −0.23° (SD 0.23°) for the whole body and −0.47° (SD 0.28°) for the lower extremities, whereas the PhA of the participants in the IG remained relatively stable at +0.01° (SD 0.20°) for the whole body and +0.08° (SD 0.37°) for the lower extremities. Analyses of variance showed large effect sizes with 43% (lower extremities) and 31.4% (whole body) explained variance among the PhA change throughout compression. As PhA is a ratio between Xc and R, it increases with the ability of cells to function as an alternating current resistor, which in turn increases with the quantity of intracellular fluid, which is a predictor of cell nutrition and functioning [49] and ultimately motor performance [50]. This can be interpreted as a meaningful positive effect of compression stockings during gaming and associated behaviors.

#### Prolonged Gaming and Associated Behaviors Lead to an Increase in ECM but Not in ICM

We found no changes in ICM among both groups but found a marginal decline of 0.2 kg in ECM among the participants in the IG and a 0.8 kg increase of ECM among the participants in the CG. With *P*=.06, the time and group interaction for ECM was at the edge of statistical significance. Further studies are needed to confirm or reject the hypothesis that an increase in TBW after prolonged gaming is mainly owing to an increase in extracellular but not intracellular fluid mass.

#### The Use of Compression Stockings Can Reduce the Decrease in PhA and the Increase in TBW (PhA* Group and TBW*Group Interactions)

Descriptive statistics and significant time and group interactions with effect sizes of 31.4% and 43% explained variance for the whole body and the lower extremities found by rmANOVAs show that compression can level out the accumulation of water after 1 day of gaming and associated behaviors. On average, the participants in the IG showed increased PhA during the study, which indicates the effectiveness of compression stockings as a preventive action during prolonged gaming. A recent study showed that periods of 3 hours of constant sitting already cause a decrease in intracellular fluid volume and increase in extracellular fluid volume, as well as a decrease in oxygenation levels without, but not with, the use of compression stockings [51]. A recent study showed that fluid accumulation in the legs grows exponentially during the first 45 minutes of seated immobility and then follows a linear function [48]. Further experimental studies on the dose–response mechanism of compression and large-scale epidemiological studies on the risk for SIT or e-thrombosis with and without compression are needed.

We think that these findings are of high relevance for public health and the prevention of future diseases in recreational gaming and the esports community. This is not only because of the increasing relevance of gaming during the recreational time of all age groups [9,10] but also for the prevention of diseases in the rising population of gaming-related professionals. The fact that prolonged sitting or standing can impair vascular function is not new. Many cases have been described in workplace settings [20-24] and also among gamers [25,29,33,35,36]. Table 6 shows a brief overview of reported clinical cases caused by prolonged gaming or computer use. A closer description of most studies can be found elsewhere [52].

**Table 6:** Examples of case studies about diseases caused by prolonged screen time.

Study	Patient description, age (years); gender	Disease	Trigger
Beasley et al [26]	32; male	Severe bilateral PE^a^	12 hours/day computer use
Ng et al [29]	12; male	DVT^b^	4 hours/day gaming
Lee [30]	24; male	Fatal PE	80 hours continuous gaming
Chew [31]	16; male	Severe PE	3-4 hours continuous gaming
Kim et al [32]	36; male	Severe PE	12 hours continuous gaming
**Elikowski et al [33]**			
	19; male	DVT	12 hours continuous gaming
	30; male	DVT	12 hours continuous computer use
	19; female	DVT	14 hours continuous gaming and computer use
	23; female	DVT	16 hours continuous computer use
	50; male	DVT	12 hours continuous computer use
	68; male	DVT	16 hours continuous computer use
Chung et al [34]	30; female	CVST^c^	≥12 hours continuous computer use
Chang et al [35]	31; male	Severe DVT	8 hours/day for 4 days continuous gaming
Braithwaite et al [36]	42; male	Severe PE	48 hours continuous gaming
**Doctor and Seth [28]**			
	50; male	Severe DVT	12 hours continuous computer use
	18; female	Severe DVT	Continuous computer use
Braithwaite et al [27]	44; male	Severe PE	36 hours continuous gaming
**Kohorst et al [25]**			
	18; male	Bilateral PE	12 hours continuous gaming
	15; male	Severe DVT and PE	4-12 hours/day continuous gaming
	13; male	PE	Continuous gaming
	17; male	DVT and severe bilateral PE	Continuous gaming

^a^PE: pulmonary embolism.

^b^DVT: deep venous thrombosis.

^c^CVST: cerebral venous sinus thrombosis.

Case studies (Table 6) and experimental studies [16,51,53] show that a single session of physical inactivity can impair vascular function. However, in a workplace setting [53] or during recreational watching of television [55], the effects are small and cases are rare. A simple explanation for this may be the fact that if we are not forced to stay physically inactive, such as physicians in surgery or travelers on an airplane, our natural behavior is to stand up and interrupt prolonged physical inactivity before damage is done to our vascular functioning. To date, we do not know to what extent this applies to highly competitive gamers and streamers. Regarding the latter, data from the web-based platform *twitch.tv* shows that not only the net revenue but also the actual viewer counts rise with the duration of a stream and that most gaming streams produce long-duration content [56,57]. At the same time, interruptions or breaks during the stream lead to a massive decline in viewership, even if the streamer just goes to the toilet for more than a minute.

In summary, studies have shown that the use of compression stockings to prevent impairments in vascular function is effective [51,58,59] and can impact physiological [60] and even psychological [61] responses to prolonged sitting or standing among healthy individuals. Our study adds that compression stockings can effectively address this issue among gamers and that BIA may be a valid tool for tracking risk factors or symptoms of diseases such as a decrease in PhA or an increase in TBW. However, the specific handling such as the duration of wearing, the optimal force of compression (eg, 15-20 mm Hg vs 20-30 mm Hg), and most importantly, a design that matches the demands of recreational and professional gamers and live streamers with usability is yet to be found. Future studies should focus on these points and the dissemination of knowledge about vascular functioning among the growing population of gamers and gaming-related live streamers.

### Strength and Limitations

As we used a field setting where we did not control for behaviors (eg, eating, drinking, breaks, and sleep), we cannot generalize the effects we found specifically to one behavior associated with gaming. An increase in TBW is ultimately mediated by water consumption and, besides being very unlikely, we cannot ignore that some participants dehydrated during the 24-hour time frame or lost water because of malnutrition; and therefore, water retention might have been underestimated in some participants. Thus, future studies should focus on underlying mechanisms, specific negative behaviors during gaming, and preventive behaviors beyond compression such as using comfortable sitting options or standardized breaks [62] and control for food and water consumption. Other factors such as stress [63] and excitement could be partly responsible for the gamers’ behavior and physical responses. However, as we used a randomized controlled design, the positive effects of the compression stockings persisted.

We analyzed the data collected during 3 consecutive years at the gaming event. Post hoc analyses showed that the analysis of only the final year data (30/50, 60% of the participants) showed results comparable with the whole sample, and as we used the same procedure and devices each year, we decided to match the data sets for this study.

BIA is a noninvasive, portable, and economic method to track the quality and quantity of body composition. The 8-electrode multifrequency BIA is considered as the gold standard among portable devices for estimating body composition in healthy populations [64]. The technique has also proven to be a valid tool to assess TBW in different body segments [65] and to estimate intracellular water (ICW) and ECW [66]. A recent study of 27 male judo athletes reported that the raw bioelectrical impedance parameters such as R, Xc, and PhA are useful in tracking fluid shifts and cell hydrations in male athletes [67]. Multifrequency BIA or bioelectrical impedance spectroscopy has been used to diagnose certain diseases that alter fluid balance [68,69]; however, reviews state that relatively large limits of agreement in estimating ICW and ECW in individual measurements limit its validity as a diagnostic instrument in single cases [70]. Nevertheless, in accordance with the study by Silva et al [67], an increasing number of recent studies report the successful use of raw segmental BIA data to track fluid changes in small samples [71] or in persons with arm and leg lymphedema [72]. BIA has been used to analyze ECW in clinical settings [73] and its usability has been proven in the assessment of lymphedema risk and its therapeutic monitoring [74]. Nevertheless, fluid distributions in the body can vary relatively quickly for various reasons. Especially among highly physically active athletes, BIA tends to have only mediocre validity and reliability in specifically assessing ICW and ECW [75]. In 2019, we also collected data for 5 kHz and 100 kHz BIA measurements to determine intracellular and extracellular fluids according to common BIA recommendations. The analysis showed similar results compared with ICM and BCM as reported in Table 4. Recent research focuses on improving the quality of segmental BIA analyses and on the distinction between ICW and ECW with the ability to track them continuously in everyday life [76]. Future studies should use these new technologies to disprove, replicate, or extend our results.

### Conclusions and Forecast

At present, an increasing number of recreational gamers professionalize themselves with competitive gaming or streaming, and for many others, the struggle for a professional career becomes their hobby. From a public health perspective, these new-formed professions and hobbies share a crucial issue: not sitting in front of the screen is ineffective in reaching your goals. This raises different questions about health issues associated with seated immobility. Different preventive measures such as designing and setting up ergonomically suitable computer workstations, using comfortable sitting positions, and avoiding long and uninterrupted physical inactivity have recently been discussed [62]. However, to the best of our knowledge, no study has described the population of live streamers and recreational or professional esports athletes as a potentially high-risk group for e-thrombosis and the possibility to effectively countermeasure this with compression stockings. We conclude from our data that the use of compression stockings during prolonged gaming and streaming lowers the risk for diseases caused by seated immobility, such as SIT, VTE, and deep venous thrombosis; however, future studies are needed to provide tailored recommendations. Finally, as there is no specific textile or clothing related to professional gaming and streaming yet, effective compression stockings may advance to a multimillion-dollar industry that favors both public health *and* the merchandise industry.

## Appendix

Multimedia Appendix 1 CONSORT-eHEALTH checklist (V 1.6.1).

## Abbreviations

BCM: body cell mass
BIA: bioelectrical impedance analysis
CG: control group
CONSORT: Consolidated Standards of Reporting Trials
ECM: extracellular body mass
ECW: extracellular water
ICM: intracellular body mass
ICW: intracellular water
IG: intervention group
PA: physical activity
PhA: phase angle
rmANOVA: repeated measurement analysis of variance
SIT: seated immobility thromboembolism
TBW: total body water
VTE: venous thromboembolism
